# Transomental hernia – An enigmatic case report causing bowel obstruction in a virgin abdomen

**DOI:** 10.1016/j.ijscr.2019.11.034

**Published:** 2019-11-27

**Authors:** Latifa Al Buainain, Kiran B. Kaundinya, Faizal N. Hammed

**Affiliations:** Bahrain Defence Force Hospital, Bahrain

**Keywords:** Transomental hernia, Bowel obstruction, Virgin abdomen, Laparoscopic repair, Case report

## Abstract

**Introduction:**

Transomental hernias are very uncommon form of internal hernias. Occasionally they may be seen in present clinical practice after bariatric surgery. Yet, this type of internal hernia remains mostly incidental and enigmatic in its presentation.

**Clinical findings:**

We present a case of transomental hernia that caused subacute small bowel obstruction in a patient with no past history of any surgical procedure, i.e. in a virgin abdomen.

**Diagnosis and therapeutic intervention:**

CT Scan of the abdomen revealed the presence of transomental hernia and it was addressed by laparoscopic surgery and an uneventful postoperative outcome.

**Conclusion:**

Transomental Hernias can be present in virgin abdomen also. Its presence should be considered in all suspicions of internal hernia occurrence even though they are commonly known to be associated with bariatric surgery, liver surgery, etc.

## Highlights

• Bowel obstruction happens to be the main presentation of most of the internal hernias.
• In its order of occourance transomental hernias are very uncommon and still are seen in present clinical practice mainly after bariatric surgery.
• We encountered a case of transomental hernia that caused subacute small bowel obstruction in a patient with no previous history of any abdominal operation, i.e. in a virgin abdomen.
• We would like to highlight the contribution of this case report to signify the presence of transomental hernias even in virgin abdomen unlike popular belief that it may occur in patients who may have undergone bariatric surgery, major liver surgery, etc.
• Whenever internal hernias are being considered as diagnosis the transomental hernias may also value consideration in a virgin abdomen.

## 1 Introduction

Internal hernias are defined as the protrusion of a viscus through a normal or an abnormal aperture within the peritoneal cavity. They are relatively uncommon clinical conditions that involve small bowel loops and occur through either congenital or acquired defects of attachment of various peritoneal folds. Subtypes include paraduodenal (53 %) and pericecal (13 %) hernias which are the most common, followed by the hernias through the foramen of Winslow (8 %), the transmesenteric (2 %) and the transomental hernias (1 %) [16].

Transomental Hernias are the rarest type of internal hernias. Occasionally congenital in a minority of paediatric cases, they are most often iatrogenic. Indeed, most transomental hernias occur as a result of previous abdominal surgery, mainly after Roux-en-Y gastric bypass. However, senile atrophy of the omentum may lead to this rare type of internal hernia, even in patients without a past surgical history.

**Fig. 1:**
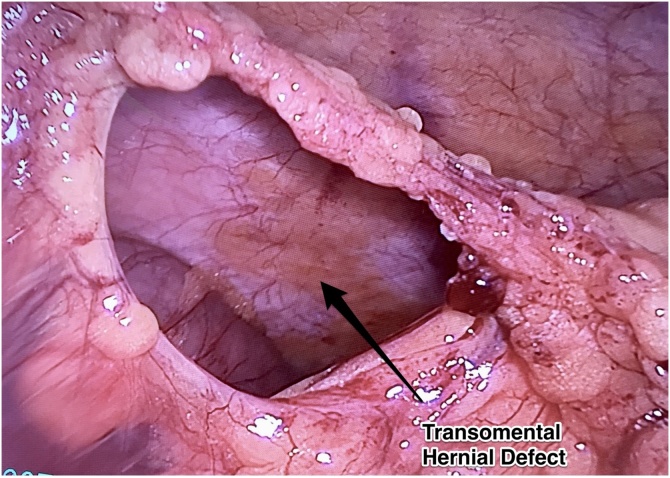
Transomental Hernial Defect - Intraoperative Finding.

Herein, we report a case of transomental hernia that presented as small bowel obstruction in a virgin abdomen and which was treated successfully in our hospital. The treating hospital is the largest military government hospital of the country and also a tertiary referral centre (Fig. 1).

This case report has been reported in line with the SCARE criteria [28].

## 2 Case presentation

A 55 year male patient came to the emergency department with complaints of sudden onset vomiting and abdominal pain since 2–3 days. The abdominal pain was generalized and colicky in nature. There were no bowel symptoms and no urinary symptoms. This was the first time he had experience this type of pain and he had no other medical or surgical history. The patient’s CT Scan of abdomen revealed incomplete small bowel obstruction. The patient had leukocytosis and neutrophilia on blood picture.

Partial obstruction not responding to bowel rest prompted surgical intervention. Laparoscopy revealed a transomental hernia with part of ileum herniating in the omental defect close to the transverse colon. The obstructed loop of small intestine in hernia was reduced and defect was deroofed. After ensuring bowel viability, the abdominal cavity was inspected for other herniae and no other site of obstruction was found. Patient was kept nil orally for 8 h and then gradually shifted to regular diet. The patient was discharged on second post operative day.

Patient was followed up in the surgical clinic after 3 weeks postoperatively and has never had similar complaints till date. The surgery was performed by the consultant general surgeon of the unit and also the first author in this case presentation.

## 3 Discussion

Openings of internal hernias can be normal (foramen of Winslow), paranormal (paraduodenal, ileocecal, supravesical fossa), or abnormal (transomental defect) with respect to the routine anatomy of the abdominal cavity [1,3]. The overall incidence of internal hernias is around 0.2 %–0.9 % [1]. Internal hernias constitute up to 0.6 %–5.8 % of all intestinal obstructions [2,9]. Hernia mortality in such instances may exceed 45 % [2,4].

Transomental Hernias(TOH) are exceptionally rare, commonly cited to account for roughly 1–4 % of all internal hernias, although this may be an underestimate [[5], [6], [7], [8], [9]]. In their series, Blachar and Federle found TOHs to constitute 5.5 % (three out of 54 cases) of internal hernias, while among the 49 internal hernia cases reviewed by Ghiassi et al., roughly 10 % (five cases) were TOHs [6,7].

Transomental hernias are generally reported in patients aged over fifty [13]. In many instances they are iatrogenic associated with surgical interventions like Roux-en-Y gastric bypass, liver transplantation, and bowel resections. They can also be secondary to trauma or peritonitis and subsequent adhesions [[10], [11], [12]].

In children, congenital transomental hernias are common. The males to female ratio is around 2 to 1 [11]. In children, disease severity depends on the orifice size and the length of the herniated small bowel loops [14]. In rare cases internal hernias through the greater or lesser omentum occur spontaneously as the result of senile atrophy without history of surgery, trauma, or inflammation [13].

Yamaguchi [15] classified transomental hernias as

- Type A (peritoneal cavity → greater omentum → peritoneal cavity),
- Type B (peritoneal cavity → omental bursa → peritoneal cavity)
- Type C (peritoneal cavity → omental bursa).

Transomental hernias are diﬃcult to diagnosis. Clinical manifestations are not specific and are similar to acute small bowel obstruction. They include nausea, vomiting, abdominal pain, distended abdomen, and constipation. Compared with other types of internal hernias, patients with transomental hernias present more frequently with strangulation of the small bowel. In most cases, a gangrenous bowel is present at the exploratory laparotomy [18]. For this reason, transomental hernias have a high postoperative mortality rate of 30 %, so emergency diagnosis and treatment are critical [[16], [17], [18]].

Abdominal CT scans are helpful for diagnosis. Abdominal CT scans may reveal dilated small bowel loops with a “beak sign,” which is a triangular configuration of the transition zone between the proximal dilated bowels and the herniated bowel segments or between dilated, herniated bowel segments and distal, collapsed bowel segments [19].

CT might also reveal a “whirl sign,” where swirling pattern of the mesenteric vessels, engorged mesenteric vessels with a large impact on the surrounding organs and bowel wall thickening are seen [20].

In addition, a transomental hernia should be suspected if the dilated small bowel loops are localized in the lesser sac (i.e., surrounded to the right by the gallbladder and liver, to the left by the stomach, and posteriorly by the pancreas) and the neck of the hernia orifice is seen as mesenteric vessels passing through the omental defect [21]. However, in most cases, a definitive diagnosis is established intraoperatively [21].

However, similar to its clinical presentation, the radiographic presentation of a TOH is non-specific and the condition is easily misdiagnosed [26,27]. In a series of 49 surgically diagnosed internal hernias, it was reported that only 16 % of preoperative CT scans were considered suspicious for an internal hernia [23].

While TOHs occur in both children and adults, they are most often seen in patients over the age of 50 years [23]. The hernial defects in pediatric cases tend to be congenital in nature. In adulthood, defects are largely iatrogenic, arising from trauma secondary to surgery, inflammation, or blunt force trauma [22,26,27]. In rare cases, hernias occur spontaneously, presumably as a result of senile atrophy of the omentum.

Internal hernias harbor a high mortality rate, because of gangrene among other complications [24,25]. As such, early diagnosis and surgical intervention are central to the treatment of these cases. Reduction of the herniated intestinal segments and resection if necrosis, perforation, or irreversible ischemia of the herniated viscera is present. In sequence, the defect of omentum must be repaired to prevent subsequent herniation [26]. If necrosis has occurred, this tissue must be excised before a repair of the defect is performed, in order to prevent further herniation.

## 4 Conclusion

Internal hernias need high index of suspicion in all cases presenting with small bowel obstruction and classical radiological signs can guide towards its diagnosis. Even without risk factors such as previous abdominal surgery, trauma or peritoneal inflammation; the possibility of bowel obstruction secondary to the internal hernia, esp. transomental hernias should be considered.

Surgical treatment based on high clinical suspicion can reduce the risk of complications and postoperative mortality in patients with a transomental hernia.

## Sources of funding

No sponsors of any kind for the case report.

## Ethical approval

Case reports exempt from ethical approval from institution.

## Consent

Written informed consent was obtained from the patient for publication of this case report and accompanying images. A copy of the written consent is available for review by the Editor-in-Chief of this journal on request

## Author contribution

1 Dr. Latifa Al Buainain - Consultant in general surgery and primary surgeon involved in the treatment of the patient’s condition. Responsible for contribution to the manuscript of the case report.
2 Dr Kaundinya Kiran B - Chief Resident in general surgery and assistant surgeon involved in the treatment of the patient’s condition. Responsible for contribution to the manuscript of the case report and corresponding author for journal submission.
3 Dr Faizal Hammed N - Chief Resident in general surgery and assistant surgeon involved in the treatment of the patient’s condition. Responsible for contribution to the manuscript of the case report.

## Registration of research studies

N.A.

## Guarantor

Dr Latifa Al Buainain - guarantor who accepts full responsibility for the work and the conduct of study, has access to the data, and controls the decision to publish.

## Provenance and peer review

Not commissioned, externally peer-reviewed

## Declaration of Competing Interest

No known conflicts of interest.
